# The role of a drug-loaded poly (lactic co-glycolic acid) (PLGA) copolymer stent in the treatment of ovarian cancer

**DOI:** 10.20892/j.issn.2095-3941.2019.0169

**Published:** 2020-02-15

**Authors:** Yanqing Wang, Xiaoyin Qiao, Xiao Yang, Mengqin Yuan, Shu Xian, Li Zhang, Dongyong Yang, Shiyi Liu, Fangfang Dai, Zhikai Tan, Yanxiang Cheng

**Affiliations:** ^1^Department of Obstetrics and Gynecology, Renmin Hospital of Wuhan University, Wuhan 430060, China; ^2^College of Biology, Hunan University, Changsha 410082, China; ^3^Department of Obstetrics and Gynecology, Renmin Hospital of Peking University, Beijing 100044, China; ^4^Department of Obstetrics and Gynecology, Taihe Hospital, Hubei University of Medicine, Shiyan 442000, China

**Keywords:** 3D printing, drug-loaded stent, local treatment, cisplatin, ovarian cancer

## Abstract

**Objectives:** Cisplatin (CDDP) is a widely used and effective basic chemotherapeutic drug for the treatment of a variety of tumors, including ovarian cancer. However, adverse side effects and acquired drug resistance are observed in the clinical application of CDDP. Identifying a mode of administration that can alleviate side effects and reduce drug resistance has become a promising strategy to solve this problem.

**Methods:** In this study, 3D printing technology was used to prepare a CDDP-poly (lactic-co-glycolic acid) (CDDP-PLGA) polymer compound stent, and its physicochemical properties and cytotoxicity were evaluated both *in vitro* and *in vivo*.

**Results:** The CDDP-PLGA stent had a significant effect on cell proliferation and apoptosis and clearly decreased the size of subcutaneous tumors in nude mice, whereas the systemic side effects were mild compared with those of intraperitoneal CDDP injection. Compared with the control group, CDDP-PLGA significantly increased the mRNA and protein levels of p-glycoprotein (*P* < 0.01; *P* < 0.01) and decreased vascular endothelial growth factor mRNA (*P* < 0.05) and protein levels (*P* < 0.01), however, CDDP-PLGA significantly decreased the mRNA and protein levels of p-glycoprotein (*P* < 0.01; *P* < 0.01) and vascular endothelial growth factor (*P* < 0.01; *P* < 0.01), which are associated with chemoresistance, in subcutaneous tumor tissue. Immunohistochemistry assay results revealed that, in the CDDP-PLGA group, the staining of the proliferation-related genes Ki67 and PCNA were lightly, and the apoptosis-related gene caspase-3 stained deeply.

**Conclusions:** PLGA biomaterials loaded with CDDP, as compared with the same amount of free CDDP, showed good efficacy in terms of cytotoxicity, as evidenced by changes in apoptosis. Continuous local CDDP release can decrease the systemic side effects of this drug and the occurrence of drug resistance and angiogenesis, and improve the therapeutic effect. This new approach may be an effective strategy for the local treatment of epithelial ovarian cancer.

## Introduction

Ovarian cancer (OC) has the highest mortality rate among gynecological tumors, and its incidence rate is increasing annually^1^. At present, the treatment for OC is mainly based on a combination of platinum-based drugs and chemotherapy plus surgery^2^. Cisplatin (CDDP) has a strong anticancer effect and is more effective in combination with other anticancer drugs than being used alone. It is currently used clinically to treat cancers such as lung cancer, bladder cancer, esophageal cancer, and gastric cancer, and it is especially useful in the treatment of testicular cancer and OC, with an initial cure rate of 100%^3–5^. However, 80% of patients eventually develop chemotherapy resistance; therefore, the prognosis of OC is extremely poor^6^. Identifying effective routes of administration in clinical practice may be a promising strategy to decrease the resistance and recurrence rate of OC.

CDDP is a potent anticancer drug known to crosslink DNA molecules in several ways, thereby interfering with mitotic cell division^7^. However, owing to its toxic effects on healthy tissues, the therapeutic potential of CDDP remains limited^8,9^. Selective delivery of CDDP to tumor cells would substantially decrease drug toxicity and improve its therapeutic index. Since the early 1970s, synthetic biodegradable polyesters such as polylactic acid/polylactide (PLA) and polyglycolic acid/polyglycolide (PGA) have been used as absorbable sutures and single silk sutures in medical applications^10,11^. Among the copolymers of PLA and PGA, poly(lactic-co-glycolic acid) (PLGA) has become an important biocompatible nontoxic polymer in drug delivery and has been approved by the US Food and Drug Administration for the treatment of various diseases, owing to its biodegradability, biocompatibility, and sustained release characteristics^10^. The degradation products of PLGA are lactic acid and glycolic acid, which are both endogenous and are simply metabolized by the body through the Krebs cycle. The use of PLGA for drug delivery or biomaterial applications is expected to have negligible systemic toxicity^9,12^. In recent years, with the advent of block copolymers, the use of PLGA as a biodegradable, nontoxic, and nonimmunogenic polymer for the development of controlled and targeted drug delivery systems has substantially increased^13^. Studies have been performed to encapsulate CDDP in dual-prepared PLGA methoxy polyethylene glycol (mPEG) nanoparticles to treat tumors^14,15^. However, PLGA nanoparticles still have a major drawback: their loading capacity is poor, and these nanoparticles exhibit highly explosive drug release, thereby resulting in a loss of drug efficacy^9,12^. Nanoparticles have poor targeting and liver first-pass effects, thus compromising the extent of their therapeutic effect.

Studies have shown that 70% of patients with OC eventually relapse despite surgery, systematic treatment, and chemotherapy^16^. Acquired multidrug resistance (MDR) is thought to develop after treatment, and recurrent OC usually does not respond to chemotherapy, thus resulting in a 5-year survival rate of < 45%^17^. One mechanism of acquired MDR is overexpression of adenosine triphosphate-binding cassette (ABC) transporters, such as p-glycoprotein (P-gp, ABCB1), which induces MDR by promoting the active efflux of chemotherapeutic agents from cells, thereby decreasing the therapeutic efficacy and allowing cells to survive^18,19^. In OC, higher P-gp expression is associated with more severe disease progression^20^.

Vascular endothelial growth factor (VEGF) is a specific endothelial cell mitogen that plays an important role in the occurrence and development of OC by signaling through specific receptors. VEGF promotes the proliferation and metastasis of OC through multiple receptor tyrosine kinase-associated pathways, and these processes are also the rate-limiting mechanisms of tumor progression^21^. In most solid tumors, high VEGF expression promotes angiogenesis in tumors and accelerates tumor growth. In OC, VEGF is associated with tumor progression and prognosis^22^; patients with high VEGF-A expression tend to have poor prognosis, and VEGF-D is associated with the metastasis of epithelial OC^23^. Proliferating cell nuclear antigen (PCNA) is an accessory protein that supports DNA replication by recruiting DNA polymerases such as polδ *via* PCNA-interacting protein (PIP)-box interactions, which play a central role in DNA damage repair mechanisms and cell proliferation^24^. Previous studies have shown an association between the poor prognosis of cancer patients and positive PCNA expression. Ki67 expression has been reported to be associated with tumor invasion and metastasis, angiogenesis, cancer cell proliferation, and poor prognosis^25^. The caspase-3 family governs the end-effect events in the apoptosis pathway, thus allowing apoptosis to be completed, and it can be used to monitor cell apoptosis.

In this study, 3D printing technology was used to enclose CDDP in a PLGA stent. The CDDP-PLGA stent, which had a high topical drug release capacity to improve bioavailability, was directly implanted near the tumor site. The stent was able to prolong the controlled release of drug and decrease the side effects of small molecule drugs without liver first-pass effects. Through this strategy, the problems of drug loading, drug burst release, and nanotoxicity caused by the aforementioned factors, as well as the preparation of nanoparticles, can be solved.

## Materials and methods

### Materials

CDDP was purchased from Med Chem Express (Cat. No. HY-17394, USA). PLGA powder was purchased from Daigang Bioengineering Co., Ltd. (Jinan, China). The OC cell line SKOV3 was obtained from the American Type Culture Collection, and the A2780 OC cell line was purchased from Nanjing Kehao Biotechnology Co., Ltd. Female BALB/c-nu nude mice 4-6 weeks of age and weighing 16–18 g were purchased from Beijing Weitong Lihua Experimental Animal Technology Co., Ltd. Primary antibodies against the following proteins were used: caspase-3 (Abcam, UK; Cat. No. ab13847; source: rabbit), Ki67 (Proteintech Group, Inc., Wuhan China; Cat. No. 27309-1-AP; source: rabbit), PCNA (Boster Biological Technology Co., Ltd., USA; Cat. No. BM0104; source: mouse); and VEGF (Abcam, UK; Cat. No. ab46154; source: rabbit). Horseradish peroxidase-labeled secondary antibodies were purchased from ASPEN (rabbit anti-goat IgG, Cat. No. AS-1108; goat anti-rabbit IgG, Cat. No. AS-1107; goat anti-mouse IgG, Cat. No. AS-1106; and goat anti-rat IgG, Cat. No. AS-1093). The ECL Developer Kit was purchased from Bio-Rad, Shanghai, China (Cat. No. 1705060). The CCK8 kit was purchased from Beyotime Biotechnology Co., Ltd., China (Cat. No. C0038).

### Preparation of the printing solution

Six milligrams of CDDP powder was dissolved in 3 mL of dimethylformamide (DMF) (Thermo Scientific™, Shanghai, China, Cat. No. 20673) and then placed on a magnetic stirrer; the sample was thoroughly dissolved to prepare a 1 mM CDDP solution, and 0.5 mL of the resulting solution was added to a dark-colored bottle. Then, 3 mL of DMF solution was added, and 0.3 g of PLGA particles (molecular weight: 500,000; PLA:PLG = 75:25) was weighed with an electronic balance, dissolved in the mixed DMF solution, and stirred for 12 h to prepare a biological 3D printing solution.

### Fabrication of the controlled-release drug stent

We pipetted 5 mL of biological 3D printing solution, which was fed into a syringe pump at a flow rate of 0.2 mL/h; the voltage applied at the injector nozzle was 2.7 kV, the collection distance was 2 mm, and a constant-temperature heating plate was added to the collection device to maintain the collection temperature at 70 °C to ensure rapid solidification of the collected stent. By controlling the computer terminal and adjusting the movement program of the collecting device to prepare brackets with different porosities according to the experimental requirements, we prepared a bracket of 150 × 150 µm. The bracket was square shaped, and the side length was 1 cm.

### Characterization of the drug-loaded stent

#### Fourier transform infrared (FTIR) spectra

FTIR spectra were recorded with KBr pellets on a Bio-Rad FTS 6000 spectrometer (Bio-Rad Company, Hercules, California, USA) at room temperature. All spectra were further processed with OPUS instrument software and were drawn in Origin 8.5.

#### Surface morphology observation of the drug-loaded stent

The drug-loaded PLGA stent was collected on foil paper, dried naturally, and observed under a field emission scanning electron microscope. Before observation, the sample surface was sprayed with gold for 60 s to enhance the conductivity of the sample. The working voltage was 5 kV, and the working distance was 8.4 mm.

### *In vitro* drug release

We weighed 20 g of anhydrous sodium chloride, 0.5 g of anhydrous potassium chloride, 3.6 g of disodium hydrogen phosphate dodecahydrate, and 0.6 g of anhydrous potassium dihydrogen phosphate with an electronic balance and added them to 1.5 mL of distilled water. HCl was used to adjust the solution pH to 7.4.

We weighed 20 mg of CDDP with an electronic balance, dissolved it in 1 mg/mL mother liquor with 20 mL of DMF solution, and accurately transferred 0.5 mL, 1 mL, 1.5 mL, and 2 mL of the mother liquor with a pipette. Mother liquor volumes of 2.5 mL and 3 mL were diluted to 10 mL with DMF, and the absorbance of the solution was measured at 266 nm with a micro-ultraviolet spectrophotometer (Thermo Scientific™, NanoDrop 2000, China).

We weighed 50 mg of the drug-loaded stent, dissolved it in 10 mL of DMF solution, and stirred the solution with a magnetic stirrer for 4 h. After the stent was completely dissolved, we collected the supernatant. The absorbance was measured with a micro-ultraviolet spectrophotometer. The drug concentration was calculated from the standard curve, and the drug amount and drug loading percentage were calculated as follows:

Drug loading rate (%) = total amount of drug in the stent/mass of the drug-loaded stent

Encapsulation rate (%) = total amount of drug in the stent/drug input amount

### Release behavior of the drug-loaded stent

We weighed thirty 5-mg capsules and placed them in 2 mL of phosphate-buffered saline (PBS), numbered them 1–30, set the constant-temperature water bath oscillator temperature to 37 °C, and set the stirring speed to 40 rpm. At 24 h, we added an equal volume of fresh PBS to each sample. Next, the sample was dried and dissolved in 10 mL of DMF solution. After the sample was completely dissolved, the amount of CDDP in solution was measured with a micro-ultraviolet spectrophotometer, and the drug concentration was calculated with a standard curve to ascertain the amounts of drug remaining in the sample and drug released per day. Five parallel data sets were required for each set of samples.

### Cell culture

The human OC cell lines SKOV3 and A2780 were cultured in complete growth medium (RPMI-1640/DMEM) containing fetal bovine serum (10%) and streptomycin (1%). Cells were maintained in a CO_2_ incubator at 37 °C, 5% CO_2_ and 98% relative humidity. Growth inhibition in different treatment groups, including the blank control, saline, PLGA, CDDP intraperitoneal injection, and CDDP-PLGA groups, was detected at different time points with CCK8 kits. Cell status was observed under an inverted microscope.

### Cytotoxicity study

The *in vitro* cytotoxicity of CDDP, CDDP-PLGA, and CDDP solution (saline) was determined with CCK8 assays. Cells were seeded (1 × 10^4^ cells per well) in 96-well plates and incubated for 24 h before the assay. Cells were treated with different concentrations of free CDDP, CDDP-PLGA or saline for 48 h. CCK8 reagent was added to each well, and the plates were incubated for 2 h at 37 °C. The absorbance was read at 490 nm with a microplate reader (BioTek, Synergy HT, USA).

Cell viability (%) = (*OD* treated − *OD* blank/*OD* control − *OD* blank) × 100%,

where *OD* treated represents the absorbance of wells containing CDDP or CDDP-PLGA, *OD* blank represents the absorbance of wells in a blank plate, and *OD* control represents the absorbance of cell culture wells containing saline.

### Cell observations

A2780 and SKOV3 cells were seeded in 48-well plates. The experiment was started when the cell density exceeded 40%. We divided each 48-well plate into a control group (saline), free CDDP group (0.35 µg/mL), and CDDP-PLGA group (5 duplicate wells per group). The total amount of CDDP released was the same in the CDDP-PLGA and CDDP groups. A2780 and SKOV3 cells were observed by microscopy after 1, 4 and 7 days of culture. Each experiment was repeated 5 times.

### Apoptosis assay

We treated A2780 and SKOV3 cells with saline, CDDP, or CDDP-PLGA. After 48 h, we digested the cells with EDTA-free trypsin, centrifuged them (300 *g*) at 4 °C for 5 min, and washed them twice with prechilled PBS. The cell pellet was resuspended in 300 µL of 1 × binding buffer. We added 5 µL of Annexin V-FITC (Tianjin Sanjian Biotechnology Co., Ltd., item number: AO2001-02P-G), mixed the sample, and incubated it for 10 min in the dark; we then added 5 µL of propidium iodide, mixed the sample, and incubated it for 5 min in the dark. Then, using a flow cytometer, we detected FITC at an excitation wavelength of 494 nm and an emission wavelength of 520 nm, and detected propidium iodide at an excitation wavelength of 493 nm and an emission wavelength of 636 nm.

### *In vivo* implantation

According to the data from the *in vitro* experiments, an antitumor experiment involving *in vivo* CDDP-PLGA stent implantation was designed. Immunodeficient athymic BALB/c nude female mice 6 weeks of age and weighing 16–18 g were cared for in accordance with protocols approved by the Institutional Animal Care and Use Committee. Subconfluent SKOV3 cells were harvested and implanted subcutaneously into the neck and back (1 × 10^7^ cells per side). When tumors reached approximately 1 cm in width, treatments were initiated. The mice were randomized to various groups receiving a total of 3 intraperitoneal injections of saline (control group) or free CDDP (6 mg/kg CDDP per dose, CDDP group), with each injection given every 3 days, or a subcutaneously implanted drug-loaded PLGA stent (CDDP-PLGA group). We collected subcutaneous tumors on the 10th day after treatment. Mouse body mass and tumor diameter were measured twice per week. The tumor volume was calculated as follows^26^: V = (a × b^2^), where a is the long diameter of the tumor, and b is the short diameter of the tumor. The mouse body mass and the tumor growth curve of each group were plotted. VEGF, PCNA, Ki67, and caspase-3 were detected by immunohistochemistry (IHC).

### IHC

The IHC procedure was performed according to the manufacturer’s instructions (DAB chromogenic Kit, Cat. No. I026-1-1 Nanjing Jiancheng Bioengineering Institute, China). We prepared 4-µm-thick paraffin-embedded tissue sections and baked the slices overnight at 62 °C. We conducted dewaxing in an alcohol gradient, and this was followed by antigen retrieval for 15 min. Endogenous peroxidase and nonspecific antibody binding were sequentially blocked with hydrogen peroxide and normal goat serum, respectively. Primary antibodies to caspase-3 (dilution 1:300), Ki67 (dilution 1:500), PCNA (dilution 1:200), and VEGF (dilution 1:100) were added to the slices for incubation overnight in a wet box at 4 °C. After equilibration at room temperature for 30 min, we added horseradish peroxidase-labeled IgG multimer secondary antibodies (dilution 1:200) and incubated the sample at room temperature for 20-30 min. We then rinsed the sample in PBS several times, added DAB coloring solution, and conducted Harris hematoxylin counterstaining. After passage through an absolute ethanol gradient, xylene dehydration, and clearing, the samples were sealed with neutral gum. The intensity of staining and the percentage of stained cells were evaluated at low magnification, and 10 fields were randomly observed.

### Western blot (WB) analysis

Total protein was extracted from tumor tissue and quantified with a BCA protein quantification kit (Beyotime Biyuntian Biotechnology Co., Ltd., China, Cat. No. P0010). A total of 10 µg of each protein sample was separated by SDS-PAGE and then transferred to a PVDF membrane, which was incubated with primary antibodies (P-gp: Absin, rabbit, 1:1000; VEGF: Abcam, ab46154, 1:1000; and GAPDH: 1:10000) at 4 °C overnight and then with secondary antibodies (horseradish peroxidase-conjugated goat anti-rabbit, ASPEN, AS1107, 1:10000) for 1 h at room temperature. Antibody reactivity was detected with an Odyssey V3.0 image scanner (Li-COR. Inc., Lincoln, NE, USA). Quantitative analysis of band intensity was conducted with ImageJ.

### Real-time PCR

Total RNA was isolated from tissues with TRIzol reagent (Invitrogen, Shanghai, China, Cat. No. 15596-026) according to the manufacturer’s instructions. cDNA synthesis was performed with a PrimeScript^TM^ RT Reagent kit with gDNA Eraser (TaKaRa). Real-time PCR (RT-PCR) was performed on a Life Technologies StepOne^TM^ RT-PCR instrument with a SYBR® Premix Ex Taq^TM^ kit (TaKaRa). Each sample was evaluated in 5 replicate wells. The reagent volumes in the reaction mixture were as follows: 5 µL 2× qPCR Mix, 1 µL primer working solution (2.5 µM), 1 µL template, 2.8 µL ddH_2_O, and 0.2 µL Rox. The relative expression levels of the test genes were calculated and normalized with the 2^-ΔΔCt^ method. The primers are shown in **Table 1**.

### Statistical analysis

The results are expressed as mean ± SD. Statistical analysis was performed with one-way analysis of variance, and Bonferroni test results with *P* < 0.05 were considered statistically significant.

## Results

### Characteristics of PLGA stents

**Figure 1:**
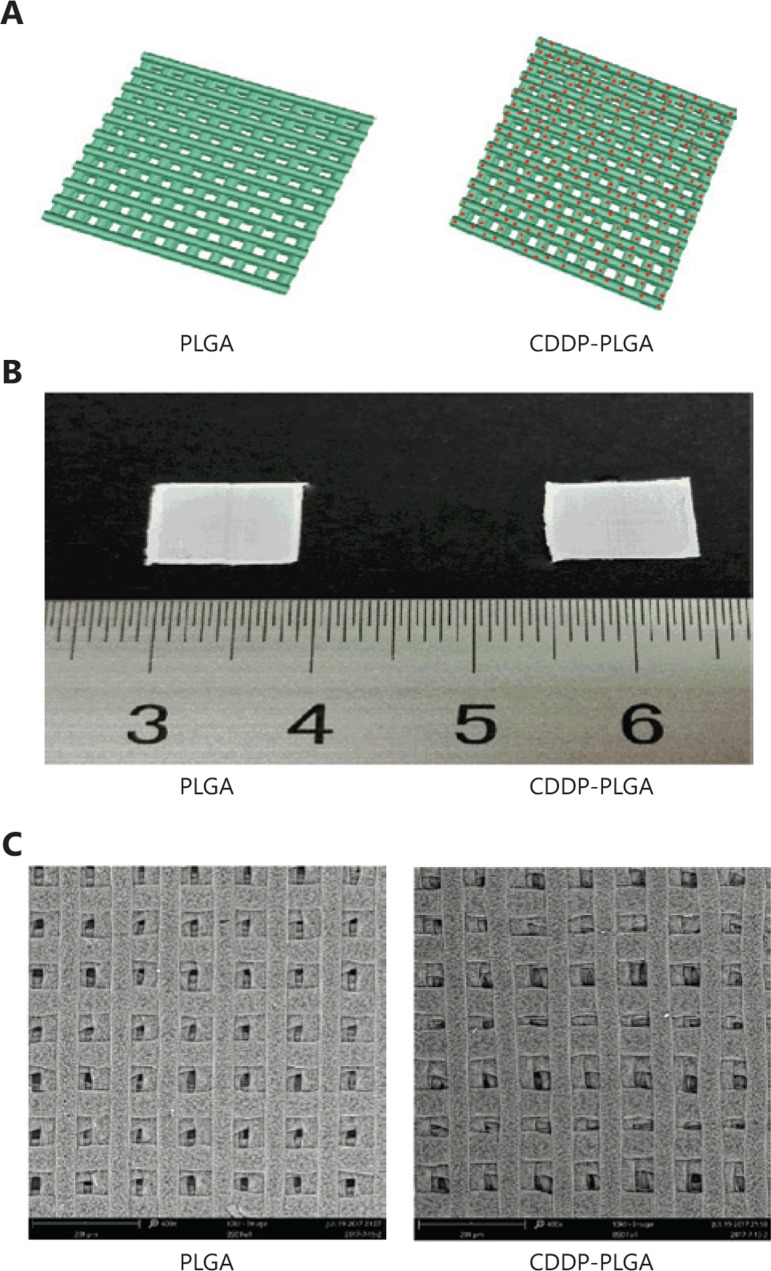
Illustration of poly(lactic-co-glycolic acid) (PLGA) stents. (A) Model diagram of a PLGA stent and a CDDP-PLGA stent. (B) Image of a PLGA stent and a CDDP-PLGA stent. (C) Scanning electron micrograph of PLGA and CDDP-PLGA.

The LA/GA ratio in PLGA polymer-loaded materials is an effective parameter for determining the custom degradation time and drug release rate. A higher GA content corresponds to a faster degradation rate^27^. **Figure 1A** shows a model diagram of an empty PLGA stent and a drug-loaded PLGA stent. The drug is evenly distributed in the stent. **Figure 1B** shows an image of the drug-loaded PLGA stent, which is square with 1-cm sides. **Figure 1C** shows a scanning electron micrograph. Because biological 3D printing enables the filament to effectively wrap CDDP, CDDP agglomeration did not appear on the surface of the stent. As shown in the figure, the similarity between the fabricated bracket and the simulated image is extremely high, thus indicating that the 3D printing device was able to accurately produce the bracket as designed.

**Figure 2:**
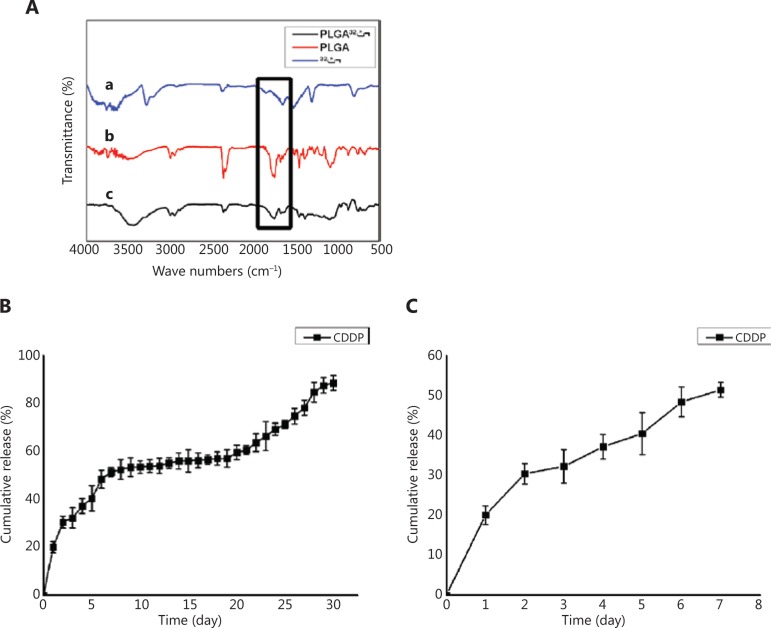
Characteristics of polymer stents. (A) FTIR spectra of (a) CDDP; (b) a PLGA stent; and (c) a CDDP-PLGA stent. (B) *In vitro* drug release profile of CDDP-PLGA (30 days). (C) *In vitro* drug release profile of CDDP-PLGA (7 days).

**Figure 2A** shows the FTIR spectra of CDDP, a PLGA stent, and a CDDP-PLGA stent. In the figure, curve a is the infrared light curve of CDDP, and the characteristic peak position of CDDP is 1,600 cm. **Curve b** shows the infrared spectrum of PLGA with a characteristic peak at 1,668 cm. **Curve c** shows the infrared spectrum of CDDP-PLGA. Two characteristic peaks appear at 1,600 cm and 1,668 cm, thus indicating that CDDP is indeed contained in the PLGA polymer material.

### *In vitro* drug release

As shown in **Figure 2B and 2C**, the sustained release curve of CDDP indicated that this compound showed a sudden release phenomenon in the initial stage (within 8 days), and the release rate reached approximately 50% in the first 7 days. However, the release rate slowed significantly after the 8th day. As shown in the figure, drug release occurred in 3 stages: the initial stage was during the first 8 days of burst release, the gentle release phase was during days 8 to 20, and the rapid release phase occurred last. In the burst phase, the PLGA carrier is immersed in PBS aqueous solution, and the PGA constituting the PLGA polymer dissolves rapidly, thus resulting in a change in the surface of the PLGA scaffold from a dense concave surface structure to a porous structure with drug incorporation in the PLGA scaffold. The drug is released quickly. In the gentle release phase, the PLGA carrier exists primarily in the form of PLA, owing to the dissolution of PGA. Because of the stability of PLA, the drug encapsulated by PLA does not reach the release rate of the first stage, and a fibrous pore structure is formed, thus enhancing the interaction between the drug and the sustained release medium, such that the drug will continue to be released slowly during this period. In the third stage, as the pores continue to increase, the stent will collapse to varying degrees. The PLA monomer is continuously released from the collapsed stent, owing to breakdown of PLA chains, thus causing the pH inside the stent to increase. An acidic pH is produced at a faster rate than the released medium can be neutralized, thereby accelerating PLGA degradation, in a phenomenon also known as autocatalysis of PLGA. Accordingly, the drug in the PLGA stent is released. The different pore sizes of the stent lead to different porosities. A larger pore size corresponds to a larger specific surface area of the stent, which in turn corresponds to a larger surface area in contact with the same volume of liquid, and the sustained release rate of the drug is proportional to the porosity.

### Effects of CDDP-PLGA stents on OC cells

**Figure 3:**
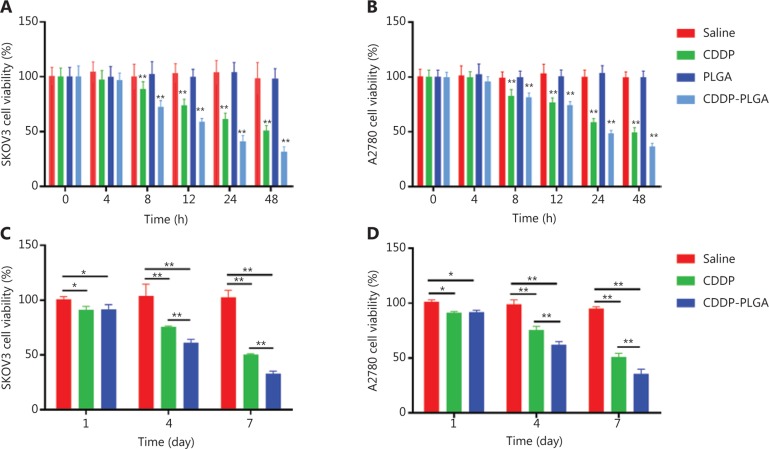
CCK8 assay for cell viability. (A) SKOV3 and (B) A2780 cell viability (%) in different treatment groups—saline, PLGA, CDDP, and CDDP-PLGA—after 0, 4, 8, 12, 24, and 48 h. (C) SKOV3 and (D) A2780 cell viability (%) in different treatment groups—saline, CDDP, and CDDP-PLGA—after 1, 4, and 7 days. *P < 0.05; **P < 0.01.

To examine the effect of CDDP-PLGA on cell proliferation, we used CCK8 assays to measure cell proliferation after 0, 4, 8, 12, 24, and 48 h. As shown in **Figures 3A** and **3B**, SKOV3 and A2780 cells in the saline, CDDP, PLGA, and CDDP-PLGA groups continued to grow, and no statistically significant difference in tumor inhibition rate was found among the 4 groups within 0-4 h (*P* > 0.05). However, after 8 h of treatment, the inhibition rate was higher in the CDDP-PLGA stent group than in the CDDP group (*P* < 0.01), and the difference in tumor inhibition increased over time. Additionally, the empty PLGA stent had no clear effect on cell proliferation.

### Cytotoxicity assay

**Figure 4:**
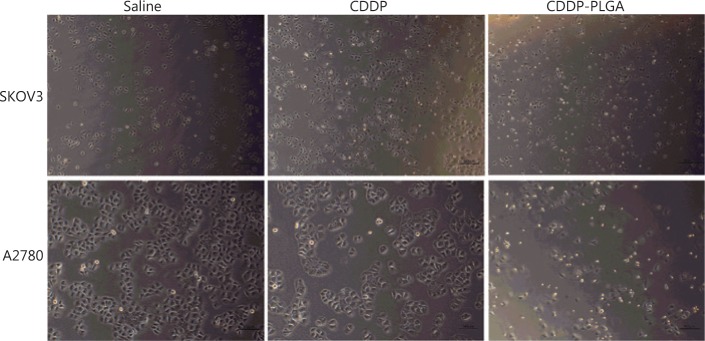
Cell morphology after 7 days, as observed under a microscope. Magnification 400×.

To determine whether the drug-loaded PLGA stent could maintain cell killing for a long time, we decreased the dosage of the drug and prolonged the administration time. SKOV3 and A2780 cell proliferation was measured after 1, 4, and 7 days with CCK8 kits. As shown in **Figures 3C** and **3D**, on days 4 and 7, cell viability was significantly inhibited in the CDDP-PLGA group (*P* < 0.01). **Figure 4** shows a micrograph of the 2 cell lines at day 4 of treatment. Compared with cells in the saline group, those in the CDDP group were rounded and fewer in number. The cells in the CDDP-PLGA group were floating, the original morphology of the cells disappeared, and the number of adherent cells decreased significantly.

### Apoptosis assay

**Figure 5:**
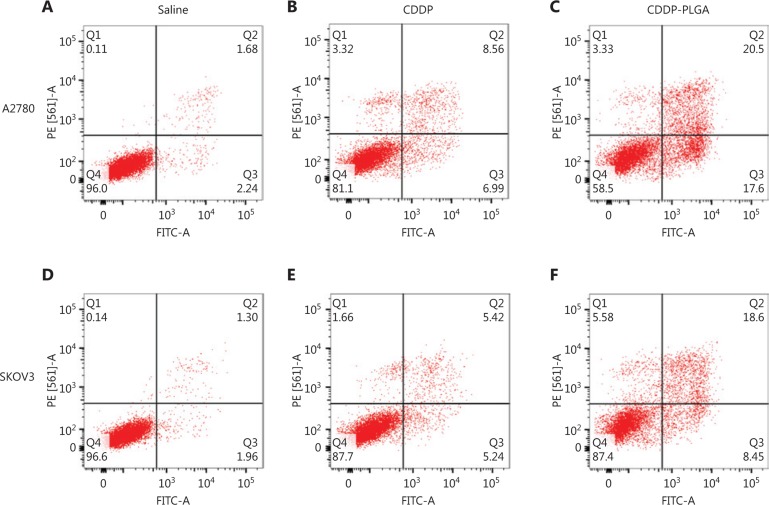
Flow cytometry analysis of SKOV3 and A2780 cells after 48 h of treatment with different drugs.

CDDP is transmitted to the cytoplasm *via* passive diffusion or a transporter; it then enters the nucleus, and acts on template strand of DNA, forming a conjugate that inhibits T7 RNA polymerase. The transmission of cellular signals is blocked, thus promoting apoptosis. We used flow cytometry to detect apoptosis in different groups of SKOV3 and A2780 cells. As shown in **Figure 5**, the effect of CDDP-PLGA on the apoptosis of OC cells was more significant than that of free CDDP (*P* < 0.01).

### *In vivo* animal experiments

**Figure 6:**
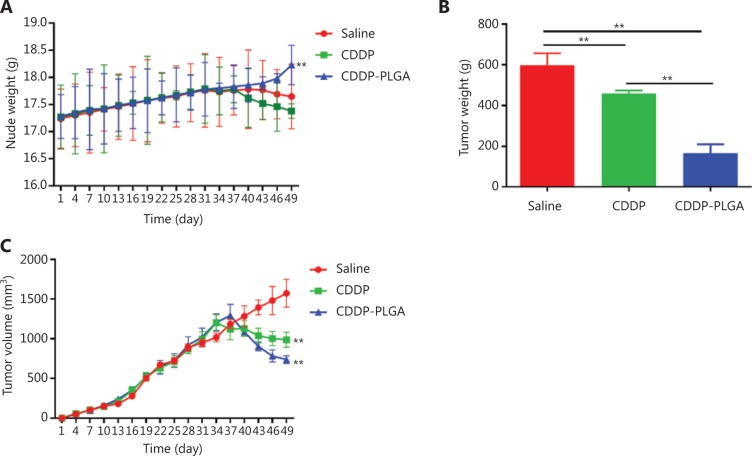
Mouse body weight and tumor volume. (A) Change in nude mouse weight during the experiment. (B) Subcutaneous tumor weight in the nude mice. (C) Change in subcutaneous tumor volume in the nude mice during the experiment. **P < 0.01.

**Figure 7:**
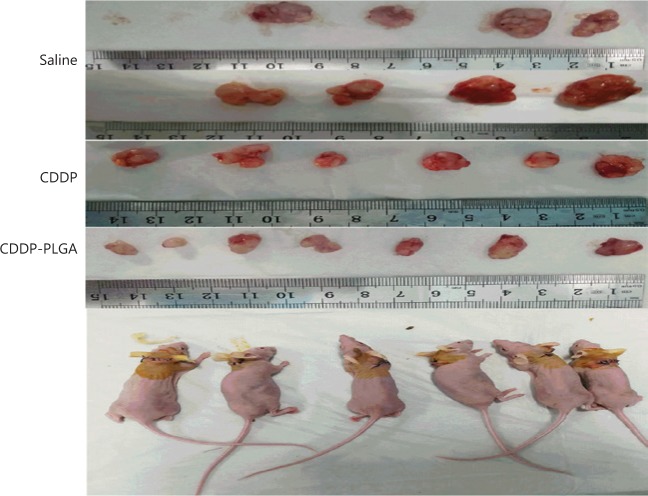
*In vivo* tumor treatment efficiency study and tumor size.

To further confirm the therapeutic effect of CDDP-PLGA on OC *in vivo*, we subcutaneously implanted SKOV3 cells in nude mice. We divided the nude mice into saline, CDDP, and CDDP-PLGA groups. **Figures 6A**, **6B**, and **6C** show the change in body weight of the nude mice during the experiment, the change in tumor volume, and the final tumor volume, respectively. As shown in **Figure 6A**, the body weight curve of nude mice in each group was recorded after tumor implantation, and the growth rate in the preneoplastic stage was relatively uniform. After drug administration on the 40th day, the side effects of CDDP-PLGA were relatively minimal; the weight of mice in the CDDP-PLGA group was not substantially reduced; and the nude mice in this group were in good condition, with rosy skin and a continued increase in body weight. In the CDDP group, because of the intraperitoneal injections of CDDP, the nude mice showed a continuous decline in weight that was more significant than that in the saline group (*P* < 0.01), and the condition of these mice was poor; they became inactive, and their skin appeared bluish-purple. **Figure 6B** shows the change in volume of the subcutaneous xenografts. Tumor volume continued to increase in the saline group and remained relatively steady in the CDDP group. The tumor volume in the CDDP-PLGA group significantly decreased, thus suggesting that CDDP-PLGA inhibits tumor growth—a favorable effect. **Figure 7** shows an image of the last tumor removed. The tumor volume in the CDDP-PLGA group was significantly less that in the control group and CDDP group (*P* < 0.01).

### Effects of the expression of cell proliferation and apoptosis related molecules in OC

**Figure 8:**
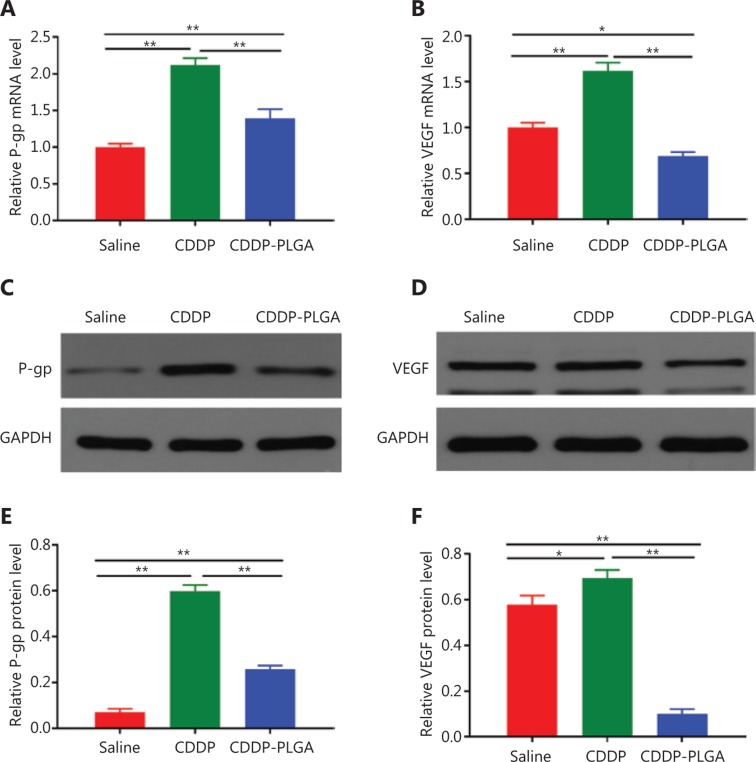
P-gp and VEGF mRNA and protein expression levels in subcutaneous tumor tissue. (A) and (B): Quantitative analysis of P-gp and VEGF by RT-PCR (*n* = 6). (C) WB analysis of P-gp and VEGF (*n* = 3). (D) and (E): Densitometric analyses of the WB bands corresponding to P-gp and VEGF. *P < 0.05; **P < 0.01.

We examined the expression levels of resistance-related genes in subcutaneous tumors from nude mice by using RT-PCR, WB, and IHC. Our results (**Figure 8**) showed that P-gp expression was significantly increased in the CDDP and CDDP-PLGA groups but not in the saline group. Large doses of CDDP have been suggested to be more likely to induce drug resistance. Angiogenesis is a multistep process that generates neovasculature in the tumor environment and is essential for tumor growth and metastasis^28^. The VEGF/VEGF receptor (VEGFR) signaling pathway is a highly promising angiogenic target, because it plays a key role in angiogenesis and tumor growth. Our results showed that VEGF protein expression was significantly higher in the CDDP group than in the normal saline group (*P* < 0.01) and was significantly lower in the CDDP-PLGA group than in the saline and CDDP groups.

**Figure 9:**
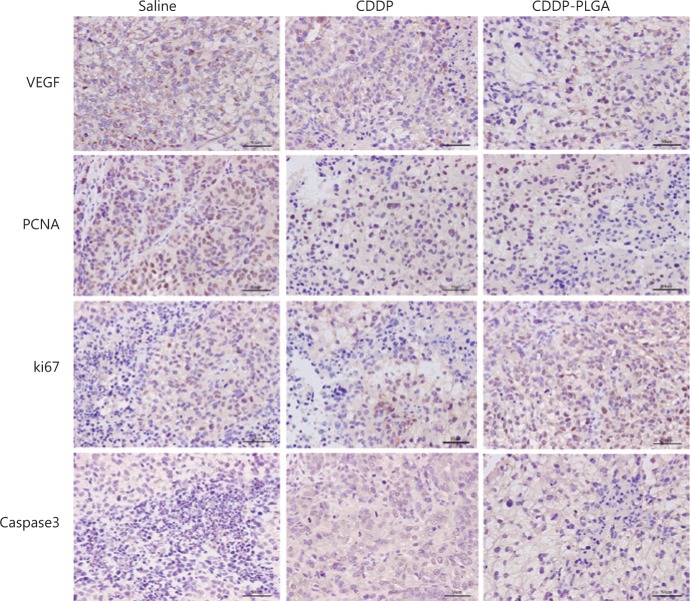
Representative IHC results. IHC analysis of VEGF, PCNA, Ki67, and Caspase-3 in tumor tissue in the saline, CDDP, and CDDP-PLGA groups. The IHC results showed that VEGF, PCNA, and Ki67 expression levels were significantly higher in the saline group than in the CDDP-PLGA group. Caspase-3 expression was lower in the saline group than in the CDDP-PLGA group (IHC staining, 400×).

We also used IHC to detect the expression of the apoptosis- and proliferation-related proteins Ki67, caspase-3, PCNA, and VEGF in tumors. The results are shown in **Figure 9**. Compared with the control and CDDP, CDDP-PLGA significantly reduced the expression levels of the angiogenesis-related factor VEGF in subcutaneous tumor tissue. Moreover, in the CDDP-PLGA group, the expression levels of the proliferation-related genes Ki67 and PCNA were decreased, and the expression level of the apoptosis-related gene caspase-3 was increased.

## Discussion

In this study, we prepared a drug-loaded scaffold with a PLGA-based vector to extend the controlled release of CDDP. Our experiments revealed that the pore size and stent affected the drug release rate. We further carried out *in vivo* and *in vitro* experiments after introducing a drug-loaded stent with a pore size of 150 × 150 µm. Among the *in vitro* assays, flow cytometry showed that CDDP-PLGA, compared with free CDDP, significantly promoted apoptosis in SKOV3 and A2780 OC cells. In nude mice, CDDP-PLGA inhibited tumor growth to a greater extent than CDDP, and the CDDP-PLGA stent group showed a significantly fewer systemic side effects than the CDDP group. The body weights of the nude mice in the CDDP-PLGA group increased throughout the experiment, and the mice were in good condition. However, the body weights of the nude mice in the CDDP group decreased significantly, and the mice had dull and cyan-colored skin and were in poor condition. These findings indicate that the PLGA polymer micelle-encapsulated drug has a high drug loading capacity, thereby improving the bioavailability and reducing the toxic side effects of this small molecule drug. In addition, because the CDDP-PLGA stent showed sustained drug release, the CDDP-PLGA group required a lower total drug dosage than the CDDP group. Our results thus suggested that drug resistance will not occur as readily in OC cells under CDDP-PLGA treatment compared with CDDP treatment alone.

CDDP activates particular signaling pathways, such as the p38, mitogen-activated protein kinase, extracellular signal-regulated protein kinase, and stress-activated protein kinase pathways, thus affecting gene expression in cells. A correlation exists between MDR proteins and CDDP resistance. In CDDP-resistant melanoma cells, MDR mRNA and protein levels are altered, and the levels of the nuclear complex decrease. MDR proteins are believed to be protective factors for cells^29^. High levels of resistance are usually caused by complex MDR mechanisms. Among them, overexpression of ABC transporters, such as P-gp, is one of the most common mechanisms^30,31^, and tumor cells have highly ordered internal resistance. P-gp, which is encoded by the MDR-1 gene, acts as a drug efflux pump that can expel a variety of chemotherapeutic drugs and decrease the accumulation of functional drugs in cells in MDR cancer, thus resulting in inefficient chemotherapy. The expression level of the most common drug-resistant protein in OC, P-gp, in subcutaneously transplanted tumors in nude mice was highest in the free CDDP group, thus suggesting a trend toward drug resistance. The development of MDR is a major obstacle to effective cancer chemotherapy. After prolonged chemotherapy, many patients experience MDR, which decreases treatment efficiency and leads to treatment failure^32,33^. PLGA is used as a drug carrier, and PLGA nanoparticles can inhibit P-gp and reverse MDR^34^.

VEGF is widely distributed in the internal and external tumor environments, and VEGF levels are significantly higher in tumor tissue than in normal tissue, thus facilitating tumor angiogenesis^35^. Angiogenesis is an important biological marker of tumor growth and malignancy. VEGF promotes the proliferation and metastasis of OC through multiple receptor tyrosine kinase-related pathways. Overexpression of VEGF in tumors has been reported to be associated with poor prognosis and is recommended as an independent predictor of patient survival^36–39^. Liu et al.^40^ have shown that treatment of prostate cancer PC3 cells with CDDP induces an increase in VEGF expression in a dose-dependent manner. In this study, VEGF protein expression was highest in the peritoneal chemotherapy group and was also high in the saline group. The expression of VEGF was low in the CDDP-PLGA stent group, thus indicating weaker tumor angiogenesis. Similar results were observed in the IHC experiments. Compared with the control and CDDP, CDDP-PLGA significantly decreased the expression levels of the angiogenesis-related factor VEGF in subcutaneous tumor tissue. Moreover, in the CDDP-PLGA group, the expression levels of the proliferation-related genes Ki67 and PCNA decreased, and the expression level of the apoptosis-related gene caspase-3 increased, thus suggesting that CDDP-PLGA can decrease the occurrence of drug resistance and angiogenesis, and improve the therapeutic effect in OC.

The application of PLGA polymer-loaded drugs in tumors is not novel. Liu et al.^41^ have used mPEG-PLGA as a material to prepare nanopolymer micelles coated with plicamycin-A (MIT-NPs) to treat BxPC-3 pancreatic cancer cells, and the MIT-NPs were effectively loaded with MIT. The loaded amount reached 96%, thus resulting in a strong *in vivo* inhibitory effect on BxPC-3 tumors in mice. He et al.^42^ have used folic acid-modified PLGA-PEG-targeting polymer micelles, paclitaxel, and CDDP for the treatment of non-small cell lung cancer and have observed good inhibition of A549 cells and A549 tumor growth in mice. However, low drug loading is a major problem in drug designs based on PLGA nanoparticles. In addition, the highly explosive drug release of nanoparticles is a major drawback. As a result, the released drug may not reach the target tissue or cells, and may result in a loss of drug efficacy. In this study, the PLGA material was fashioned into a stent that was surgically implanted near the tumor site; this approach not only circumvented the first-pass effect of traditional administration *in vivo* but also substantially decreased the systemic side effects of the chemotherapy drug. In addition, because the production parameters of drug-loaded PLGA stents can be adjusted according to the amount of drug required by different patients, thus enabling controlled release of chemotherapeutic drugs, the drug can be maintained within the therapeutic range for a long time, and fluctuations in drug concentration caused by repeated administration can be minimized. Direct drug administration leads to unstable drug concentrations that last only a short period; consequently, repeated drug administration is required, which may cause physical and mental pain in patients. PLGA is a polymer material that plays an important role in controlled drug release and targeted drug delivery. This study focused on the controlled drug release capacity of PLGA membranes, which can circumvent certain disadvantages of nanoparticle administration. For example, nanoparticles are easily phagocytized by the human immune system, and tissue or cell targeting is not sufficiently strong. Excessive modification of an encapsulated drug causes a decrease in therapeutic potential, owing to accelerated release; consequently, long-term sustained release is not achieved. Therefore, we created a biodegradable CDDP-PLGA stent that can be directly applied during OC surgery in clinical practice, thus enabling drug release at the target position and consequently preventing OC recurrence.

## Conclusions

In this study, we successfully prepared a drug-loaded stent providing sustained drug release for the treatment of OC. On the basis of the characteristics of PLGA gradient degradation, a bioactive 3D printing device was used to prepare the drug-loaded stent coated with the chemotherapeutic drug CDDP. The advantages of drug-loaded stents compared with intraperitoneal drug injection were demonstrated in *in vitro* and *in vivo* experiments. The drug-loaded stent achieved better tumor suppression at a relatively low dose. The degradation products of PLGA are carbon dioxide and water, which can be directly absorbed by the body, thus avoiding secondary injury after surgery. This study provides a new technical means for tumor interventional therapy.

## Figures and Tables

**Table 1:** Primer sequences

Gene	Primer sequence
GAPDH	Forward 5'-TGAAGGGTGGAGCCAAAAG-3'
	Reverse 5'-AGTCTTCTGGGTGGCAGTGAT-3'
P-gp	Forward 5'-CATTGCGATAGCAGGAGTGGT-3'
	Reverse 5'-CACCAAGTAGGCACCGAACC-3'
VEGF	Forward 5'-GCTACTGCCGTCCGATTGAG-3'
	Reverse 5'-GGCTTTGTTCTGTCTTTCTTTGGT-3'

## References

[1] Torre LA, Trabert B, DeSantis CE, Miller KD, Samimi G, Runowicz CD (2018). Ovarian cancer statistics, 2018.. CA Cancer J Clin..

[2] Ferrandina G, Corrado G (2018). Treatment of platinum refractory or resistant ovarian cancer.. Lancet Oncol..

[3] Galluzzi L, Vitale I, Michels J, Brenner C, Szabadkai G, Harel-Bellan A (2014). Systems biology of cisplatin resistance: past, present and future.. Cell Death Dis..

[4] Oberoi HS, Nukolova NV, Kabanov AV, Bronich TK (2013). Nanocarriers for delivery of platinum anticancer drugs.. Adv Drug Deliv Rev..

[5] Seiler R, Oo HZ, Tortora D, Clausen TM, Wang CK, Kumar G (2017). An oncofetal glycosaminoglycan modification provides therapeutic access to cisplatin-resistant bladder cancer.. Eur Urol..

[6] Christian J, Thomas H (2001). Ovarian cancer chemotherapy.. Cancer Treat Rev..

[7] Chen P, Li J, Chen YC, Qian H, Chen YJ, Su JY (2016). The functional status of DNA repair pathways determines the sensitization effect to cisplatin in non-small cell lung cancer cells.. Cell Oncol (Dordr)..

[8] Oberoi HS, Nukolova NV, Laquer FC, Poluektova LY, Huang J, Alnouti Y (2012). Cisplatin-loaded core cross-linked micelles: comparative pharmacokinetics, antitumor activity, and toxicity in mice.. Int J Nanomed..

[9] Kumari A, Yadav SK, Yadav SC (2010). Biodegradable polymeric nanoparticles based drug delivery systems.. Colloids Surf B: Biointerfaces..

[10] Astete CE, Sabliov CM (2006). Synthesis and characterization of PLGA nanoparticles.. J Biomater Sci Polym Ed..

[11] Arasoglu T, Derman S, Mansuroglu B, Yelkenci G, Kocyigit B, Gumus B (2017). Synthesis, characterization and antibacterial activity of juglone encapsulated PLGA nanoparticles.. J Appl Microbiol..

[12] Danhier F, Ansorena E, Silva JM, Coco R, Le Breton A, Preat V (2012). PLGA-based nanoparticles: an overview of biomedical applications.. J Control Release..

[13] Sadat Tabatabaei Mirakabad F, Nejati-Koshki K, Akbarzadeh A, Yamchi MR, Milani M, Zarghami N (2014). PLGA-based nanoparticles as cancer drug delivery systems.. Asian Pac J Cancer Prev..

[14] Liu P, Sun Y, Wang Q, Sun Y, Li H, Duan Y (2014). Intracellular trafficking and cellular uptake mechanism of mPEG-PLGA-PLL and mPEG-PLGA-PLL-Gal nanoparticles for targeted delivery to hepatomas.. Biomaterials..

[15] Cai C, Xie Y, Wu L, Chen X, Liu H, Zhou Y (2017). PLGA-based dual targeted nanoparticles enhance miRNA transfection efficiency in hepatic carcinoma.. Sci Rep..

[16] Fung-Kee-Fung M, Oliver T, Elit L, Oza A, Hirte HW, Bryson P (2007). Optimal chemotherapy treatment for women with recurrent ovarian cancer.. Curr Oncol..

[17] Buys TP, Chari R, Lee EH, Zhang M, MacAulay C, Lam S (2007). Genetic changes in the evolution of multidrug resistance for cultured human ovarian cancer cells.. Genes Chromosomes Canc..

[18] Wang B, Li S, Meng X, Shang H, Guan Y (2015). Inhibition of mdr1 by G-quadruplex oligonucleotides and reversal of paclitaxel resistance in human ovarian cancer cells.. Tumour Biol..

[19] Glavinas H, Krajcsi P, Cserepes J, Sarkadi B (2004). The role of ABC transporters in drug resistance, metabolism and toxicity.. Curr Drug Deliv..

[20] Penson RT, Oliva E, Skates SJ, Glyptis T, Fuller AF,  Goodman A (2004). Expression of multidrug resistance-1 protein inversely correlates with paclitaxel response and survival in ovarian cancer patients: a study in serial samples.. Gynecol Oncol..

[21] Funakoshi T, Latif A, Galsky MD (2014). Safety and efficacy of addition of VEGFR and EGFR-family oral small-molecule tyrosine kinase inhibitors to cytotoxic chemotherapy in solid cancers: a systematic review and meta-analysis of randomized controlled trials.. Cancer Treat Rev..

[22] Cheng D, Liang B, Li Y (2013). Serum vascular endothelial growth factor (VEGF-C) as a diagnostic and prognostic marker in patients with ovarian cancer.. PLoS One..

[23] He L, He J, Zhao X (2016). Expression of VEGF-D in epithelial ovarian cancer and its relationship to lymphatic metastasis.. Asia Pac J Clin Oncol..

[24] Han YH, Gao B, Huang JH, Wang Z, Guo Z, Jie Q (2015). Expression of CD147, PCNA, VEGF, MMPs and their clinical significance in the giant cell tumor of bones.. Int J Clin Exp Pathol..

[25] Kamal CK, Simionescu CE, Margaritescu C, Stepan A (2012). P53 and Ki67 immunoexpression in mucinous malignant ovarian tumors.. Rom J Morphol Embryol..

[26] Weissleder R (2006). Molecular imaging in cancer.. Science..

[27] Xu Y, Kim CS, Saylor DM, Koo D (2017). Polymer degradation and drug delivery in PLGA-based drug-polymer applications: a review of experiments and theories.. J Biomed Mater Res B Appl Biomater..

[28] Shaili E (2014). Platinum anticancer drugs and photochemotherapeutic agents: recent advances and future developments.. Sci Prog..

[29] Fojo AT, Ueda K, Slamon DJ, Poplack DG, Gottesman MM, Pastan I (1987). Expression of a multidrug-resistance gene in human tumors and tissues.. Proc Natl Acad Sci USA..

[30] Kohno K, Sato S, Takano H, Matsuo K, Kuwano M (1989). The direct activation of human multidrug resistance gene (MDR1) by anticancer agents.. Biochem Biophys Res Commun..

[31] Lee SH, Jeong D, Han YS, Baek MJ (2015). Pivotal role of vascular endothelial growth factor pathway in tumor angiogenesis.. Ann Surg Treat Res..

[32] Szakacs G, Paterson JK, Ludwig JA, Booth-Genthe C, Gottesman MM (2006). Targeting multidrug resistance in cancer.. Nat Rev Drug Discov..

[33] Li B, Xu H, Li Z, Yao M, Xie M, Shen H (2012). Bypassing multidrug resistance in human breast cancer cells with lipid/polymer particle assemblies.. Int J Nanomed..

[34] Sahoo SK, Labhasetwar V (2005). Enhanced antiproliferative activity of transferrin-conjugated paclitaxel-loaded nanoparticles is mediated via sustained intracellular drug retention.. Mol Pharm..

[35] Baker EA, Bergin FG, Leaper DJ (2000). Plasminogen activator system, vascular endothelial growth factor, and colorectal cancer progression.. Mol Pathol..

[36] Hartenbach EM, Olson TA, Goswitz JJ, Mohanraj D, Twiggs LB, Carson LF (1997). Vascular endothelial growth factor (VEGF) expression and survival in human epithelial ovarian carcinomas.. Cancer Lett..

[37] Paley PJ, Staskus KA, Gebhard K, Mohanraj D, Twiggs LB, Carson LF (1997). Vascular endothelial growth factor expression in early stage ovarian carcinoma.. Cancer..

[38] Kassim SK, El-Salahy EM, Fayed ST, Helal SA, Helal T, Azzam Eel D (2004). Vascular endothelial growth factor and interleukin-8 are associated with poor prognosis in epithelial ovarian cancer patients.. Clin Biochem..

[39] Siddiqui GK, Elmasry K, Wong Te Fong AC, Perrett C, Morris R, Crow JC (2010). Prognostic significance of intratumoral vascular endothelial growth factor as a marker of tumour angiogenesis in epithelial ovarian cancer.. Eur J Gynaecol Oncol..

[40] Liu F, Wang J, Fu Q, Zhang X, Wang Y, Liu J (2015). VEGF-activated miR-144 regulates autophagic survival of prostate cancer cells against cisplatin.. Tumour Biol..

[41] Liu XJ, Li L, Liu XJ, Li Y, Zhao CY, Wang RQ (2017). Mithramycin-loaded mPEG-PLGA nanoparticles exert potent antitumor efficacy against pancreatic carcinoma.. Int J Nanomed..

[42] He Z, Huang J, Xu Y, Zhang X, Teng Y, Huang C (2015). Co-delivery of cisplatin and paclitaxel by folic acid conjugated amphiphilic PEG-PLGA copolymer nanoparticles for the treatment of non-small lung cancer.. Oncotarget..

